# Effects of mechanical strain on periodontal ligament fibroblasts in presence of *Aggregatibacter actinomycetemcomitans* lysate

**DOI:** 10.1186/s12903-021-01761-3

**Published:** 2021-08-18

**Authors:** Agnes Schröder, Julia Stumpf, Eva Paddenberg, Patrick Neubert, Valentin Schatz, Josef Köstler, Jonathan Jantsch, James Deschner, Peter Proff, Christian Kirschneck

**Affiliations:** 1 Department of Orthodontics, University Hospital Regensburg, Regensburg, Germany; 2 Institute of Clinical Microbiology and Hygiene, University Hospital Regensburg, Regensburg, Germany; 3 Department of Periodontology and Operative Medicine, University Medicine Mainz, 55131 Mainz, Germany

**Keywords:** Periodontal ligament fibroblast, PDL, Orthodontic tooth movement, Periodontitis, Inflammation

## Abstract

**Purpose:**

Many adult orthodontic patients suffer from periodontitis, which is caused by oral pathogens such as the gram-negative *Aggregatibacter actinomycetemcomitans* (*Agac*). Like orthodontic tooth movement, periodontitis is associated with inflammation and alveolar bone remodelling thereby affecting orthodontic treatment. Interactions of both processes, however, are not sufficiently explored, particularly with regard to oxidative stress.

**Methods:**

After preincubation with *Agac* lysate for 24 h periodontal ligament fibroblasts (PDLF) were either stretched or compressed for further 48 h simulating orthodontic forces in vitro. We analysed the expression of genes and proteins involved in the formation of reactive oxygen species (NOX-4, ROS) and nitric oxide (NOS-2), inflammation (*TNF, IL-6, PTGS-2*) and bone remodelling (*OPG, RANKL*).

**Results:**

*Agac* lysate elevated the expression of NOX-4, NOS-2, inflammatory *IL-6* and *PTGS-2* and the bone-remodelling *RANKL/OPG* ratio during compressive, but not tensile mechanical strain. *Agac* lysate stimulated pressure-induced inflammatory signalling, whereas surprisingly ROS formation was reduced. Pressure-induced downregulation of *OPG* expression was inhibited by *Agac* lysate.

**Conclusions:**

*Agac* lysate impact on the expression of genes and proteins involved in inflammation and bone remodelling as well as ROS formation, when PDLF were subjected to mechanical forces occurring during orthodontic tooth movement.

**Supplementary Information:**

The online version contains supplementary material available at 10.1186/s12903-021-01761-3.

## Introduction

Orthodontic treatment prevents and corrects dysfunctions, malocclusions and jaw malposition. In addition to essential skills like speaking or chewing, oral health is considered an essential foundation of holistic health including physical and mental well-being, contributing to the quality of life [1, 2]. Oral diseases are one of the most common public health conditions and remain a major global health burden [2]. Among these, dental caries and periodontal disease are cited by the WHO (world health organization) as the most important oral health burdens worldwide [3, 4]. Increased plaque accumulation favours the development of pathogenic processes in the periodontium and is associated with gingivitis and also periodontitis [5]. Periodontitis is characterised by a bacterial inflammatory pathophysiological breakdown of periodontal tissue with irreversible bone loss [6]. Therefore it has the potential to interfere with orthodontic therapy. An uncontrolled inflammatory process during orthodontic treatment in a periodontally challenged dentition can result in orthodontically induced root resorption, including destruction of the periodontal tissue [7]. In contrast to the pathophysiological inflammation and destruction of periodontal tissue that can be detected as signs and symptoms of periodontitis [6, 8], a local so-called controlled sterile inflammatory cascade is activated during orthodontic tooth movement inducing bone remodelling [7, 9]. During orthodontic treatment, cells in the periodontal ligament are subjected to compressive and tensile strain [7, 9, 10], resulting in bone deposition by osteoblasts at tension and bone resorption by osteoclasts at pressure areas [11]. Osteoclastogenesis is controlled by the expression of receptor activator of NF-kB ligand (RANKL) and its decoy receptor osteoprotegerin (OPG) [12]. Next to osteoblasts, periodontal ligament fibroblasts (PDLF) can express both genes and thereby modulate differentiation of precursor cells to bone-resorbing osteoclasts in response to mechanical stress [10, 13]. In a healthy periodontal condition, orthodontic treatment does not induce gingival recessions, alveolar bone loss or increased pocket depths to a clinically relevant extent [14], whereas this is true for orthodontic treatment during active periodontitis [15].

Periodontitis is a complex clinical entity, determined by the nature of the biofilm, individual risk factors and host defence [16]. In this respect, the importance of bacterial colonisation for the planning and implementation of orthodontic therapy cannot be neglected and of clinical relevance due to the increasing number of adult orthodontic treatments [17]. The gram-negative periodontal pathogens, *Aggregatibacter actinomycetemcomitans* (*A. actinomycetemcomitans, Agac*) and *Porphyromonas gingivalis* (*P. gingivalis*) possess virulence factors, which can activate neutrophil granulocytes, macrophages and fibroblasts [6]. When stimulated with the lipopolysaccharide of *P. gingivalis* and *A. actinomycetemcomitans,* human PDLF showed a significantly increased expression of proinflammatory cytokines such as IL-6 and TNF [18]. As similar effects occur during orthodontic force application [10], it can be assumed that bacterial stress may influence orthodontic treatment. Reactive oxygen and nitrogen species (ROS/RNS) are increasingly generated by neutrophil granulocytes and fibroblasts in the course of inflammatory processes such as periodontitis [19]. In addition, reactive oxygen metabolites and nitric oxide are produced by macrophages and neutrophil granulocytes to defend against bacteria and kill germs [20]. As ROS are also produced intracellularly under oxygen-deficient conditions during different enzymatically modulated reactions, it is conceivable that hypoxia occurring during tooth movement [21] could stimulate PDLF to produce ROS. The key interacting enzyme in the so-called oxidative burst, NADPH oxidase (NOX), forms large amounts of ROS [22]. The objective of this study was thus to determine the effects of *A. actinomycetemcomitans (Agac)* lysate on the expression of inflammatory and bone-remodelling genes in PDLF during mechanical loading, in particular with regard to ROS production.

## Material and methods

### Isolation of human periodontal ligament fibroblasts

For the experiments we pooled periodontal ligament fibroblasts (PDLF) from six individual patients (three males, three females) aged 17–27 years. Isolation of periodontal tissue from intact third molars without any carious lesions after extraction for medical reasons and the subsequent experiments were approved by the ethics committee of the University of Regensburg, Germany (Approval Number 12-170-0150). Informed written consent was obtained from the subjects. To isolate PDLF, periodontal ligament was removed from the middle third of the tooth root under sterile conditions and transferred to 5 ml sterile PBS (14190094, Life Technologies) containing 1% antibiotic/antimycotic (AA, with 10,000 units/ml penicillin, 10 mg/ml streptomycin and 25 μg/ml amphotericin B; A5955, Sigma-Aldrich). After addition of 8 µl collagenase type II (~ 160 U/ml; 17101-015, Thermo Fisher Scientific), the periodontal ligament was incubated for 20 min at 37 °C. The supernatant was removed, the isolated tissue washed again with sterile PBS + AA and the tissue pieces were placed in 6-well cell culture plates (353046, Omnilab/BD) containing 2 ml DMEM High Glucose (D5671, Sigma-Aldrich) with 10% fetal bovine serum (FBS, P30-3302, PAN-Biotech), 1% AA (A5955, Sigma-Aldrich); 1% L-glutamine (G7513, Sigma-Aldrich) per well and cultured under cell culture conditions until fibroblasts grew out (Additional file 1: Figure S1).

### In vitro cell culture experiments

For experiments, we seeded 70,000 PDLF up to the sixth passage either on polystyrol plates (353046, BD Bioscience) for pressure application or on collagen-coated bioflex plates (BF-3001C, Dunn Labortechnik) for tensile strain in DMEM High Glucose (D5671, Sigma Aldrich), supplemented with 10% FBS (P30-3302, PAN Biotech), 1% AA (A5955, Sigma Aldrich), 1% L-glutamine (G7513, Sigma Aldrich) and 1% ascorbic acid (B6891, Sigma Aldrich). We either left the cells untreated or added 200 µl *A. actinomycetemcomitans* (*Agac*) lysate for 24 h [23]. After that we compressed PDLF with a sterile glass plate (2 g/cm^2^) [24] or stretched them using a spherical silicone stamp (16%) [25] for another 48 h according to validated and published protocols without changing the cell culture medium.

### Preparation of *Agac* lysate

*Aggregatibacter actinomycetemcomitans* (DSM 11123; provided by the German Collection of Microorganisms and Cell Cultures) was incubated anaerobically on sheep blood agar plates for 3–5 days at 37 °C. A single colony was used to inoculate a brain–heart infusion broth (Carl Roth). When the bacteria attained post-logarithmic growth, they were harvested by centrifugation and concentrated to an optical density of 1 at 690 nm equivalent to ≈ 1 × 10^8^ colony-forming units per ml. The lysate was prepared by heating the suspension for 10 min at 60 °C.

#### RNA isolation

For RNA isolation, we vortexed PDLF in 500 µl Trizol (30-2010, VWR) with 100 µl chloroform (1.02445.1000, VWR) for 30 s. After 15 min incubation on ice, the suspension was centrifuged at 13,000 rpm for 15 min at 4 °C and the supernatant was mixed with 500 μl of pre-cooled isopropanol (20.842.330, VWR). The samples were mixed by inverting and incubated overnight at − 80 °C. Samples were centrifuged at 13,000 rpm at 4 °C for 30 min and washed twice with 80% ethanol (1.00983, Sigma-Aldrich). The pellet was dried for at least 30 min and resuspended in 20 μl RNase-free water (T143.5, Carl Roth). The concentration of RNA was measured in the nano-photometer NP60 (Implen).

#### cDNA synthesis

To minimize variations, we prepared a master mix consisting of 200 U M-MLV reverse transcriptase (M1705, Promega), 40 nmol dNTPs (L785.2, Carl Roth), 0.1 nmol Oligo (dT) 18-primer (SO132, Thermo Fisher), 0.1 nmol random hexamer primer (SO142, Thermo Fisher), 40 U RiboLock RNase Inhibitor (EO0382, Thermo Fisher) and 1× M-MLV RT Buffer (M1705, Promega) and mixed the master-mix with equal amounts of RNA (100 ng). Samples were incubated at 37 °C for 1 h, followed by 95 °C for 2 min for transcriptase inactivation in a thermocycler (Doppio 2.48 well Block, VWR).

#### Quantitative real-time polymerase chain reaction (RT-qPCR)

To determine the relative expression of the examined genes, an individual primer mix containing 7.5 µl SYBR®Green JumpStart™ Taq ReadyMix™ (S4438, Sigma–Aldrich), the respective primer pair (7.5 pmol, 0.75 µl—3.75 pmol/primer) and 5.25 µl nuclease-free H_2_O (T143, Carl-Roth GmbH) were mixed with 1.5 µl cDNA per sample in a 96-well TW-MT plate (712282, Biozym). RT-qPCR was performed in a realplex2 master cycler (Eppendorf). The plates were heated to 95 °C. for 5 min followed by 45 cycles at 95 °C for 10 s, at 60 °C for 8 s and at 72 °C for 8 s. For compression experiments we used a combination of *PPIB/RPL22* [23] as reference genes for normalisation of target gene expression, while a combination of *TBP/PPIB* [25] was used for tensile strain experiments (Table 1). We determined the used reference gene combination as most stable reference combination for the tested setup based on mathematical algorithms.

**Table 1:** RT-qPCR primer sequences for reference genes (*TBP, RPL22, PPIB*) and target genes

Gene symbol	Gene name	5′-forward primer-3′	5′-reverse primer-3′
*PPIB*	Peptidylprolyl isomerase A	TTCCATCGTGTAATCAAGGACTTC	GCTCACCGTAGATGCTCTTTC
*RPL22*	Ribosomal protein L22	TGATTGCACCCACCCTGTAG	GGTTCCCAGCTTTTCCGTTC
*IL-6*	Interleukin-6	TGGCAGAAAACAACCTGAACC	CCTCAAACTCCAAAAGACCAGTG
*PTGS-2*	Prostaglandin-endoperoxide synthase-2	GAGCAGGCAGATGAAATACCAGTC	TGTCACCATAGAGTGCTTCCAAC
*TBP*	TATA binding protein	CGGCTGTTTAACTTCGCTTCC	TGGGTTATCTTCACACGCCAAG
*TNF*	Tumor necrose factor	GAGGCCAAGCCCTGGTATG	CGGGCCGATTGATCTCAGC
*OPG*	Osteoprotegerin	TGTCTTTGGTCTCCTGCTAACTC	ACGCTCCAGGACTTATACCG
*RANKL*	Receptor activator of NFkB ligand	ATACCCTGATGAAAGGAGGA	GGGGCTCAATCTATATCTCG
*NOX-4*	NADPH oxidase 4	TCTGCCTGTTCATCTGGCTCTCCA	AGCCAAGAGTGTTCGGCACATGGGTA

Relative gene expression was calculated using the formula 2^−ΔCq^ [26], where ∆Cq was calculated from Cq (target gene)—Cq (geomean reference genes). It was then divided by the arithmetic mean of the control group in order to obtain a relative gene expression in relation to the control group.

#### Protein isolation and determination of concentration

For protein isolation PDLF were scraped off in 100 µl CelLytic M (C2978, Sigma Aldrich) with protease inhibitors diluted 1:100 according to manufacturer’s instructions (87786, Thermo Fisher) and placed on ice for 15 min. Samples were centrifuged at 13,000 rpm for 10 min at 4 °C. Protein concentration was determined with RotiQuant (K015.3, Carl Roth).

#### Western Blot analysis

Equal amounts of protein (10 µg) were heated to 70 °C for 7 min with 1× loading buffer (6× sample buffer: of 0.375 M Tris/HCl pH6.8, 30% glycerol (3783.1, Carl Roth), 12% SDS (8029.1, Carl Roth), 0.6% bromophenol blue (T116.2, Carl Roth), 1% dithiothreitol (6908.1, Carl Roth)). After centrifugation (7000 rpm for 7 min at 4 °C), the samples were stored at − 80 °C for further use. Proteins were separated on polyacryl amid gels consisting of a 10% separation gel (38% H_2_O_d_, 34% acrylamide mix/rotiphoresis gel 30 (3029.1, Carl Roth), 26% 1.5 M Tris/HCl pH8.8, 1% SDS (8029.1, Carl Roth), 1% ammonium peroxide sulphate (9592.2 Carl Roth), 0.04% TEMED (T9281, Sigma Aldrich)) and a collecting gel (70% H_2_O_d_, 16.5% acrylamide mix/rotiphoresis gel 30, 12.5% 1.0 M Tris/HCl pH6.8, 1% SDS, 1% APS and 0.1% TEMED) using the Mini-PROTEAN Tetra Cell (1658005EDU, BIO-RAD) in 1× electrode buffer (10× electrode buffer: 24.8 mM Tris/Trizma base (T1503, Sigma Aldrich), 3.5 mM SDS (8029.1, Carl Roth), 1.9 M glycine (33226, Sigma-Aldrich)). Protein standard (5 µl; 26,616, Thermo Fisher) was pipetted onto each gel. The gels were first connected for 30 min at 80 V, then left to run for 90 min at 120 V. Afterwards, proteins were transferred onto a PVDF membrane (T830.1, Carl Roth) at 90 V for 90 min in 1× transfer buffer (20× transfer buffer: 0.2 M Tris/Trizma base (T1503, Sigma Aldrich), 2 M glycine (33226, Sigma-Aldrich)) including 10% methanol using a tank-blot apparature (1660827EDU, BIO-RAD). The membrane was blocked for 1 h at RT in 5% milk (T145.3, Carl Roth) in TBS-T. The membranes were incubated over night at 4 °C (NOS-2: 1:2000, PA3-030A, invitrogen; NOX4: 1:2000; PA5-88106, invitrogen; ACTIN: 1:3000, ABIN 274248, antibody-online) in 5% milk (T145.3, Carl Roth) in TBS-T with agitation. After washing three times in TBS-T, membranes were incubated with the secondary antibody (1:5000: 611–1302, RockLand) in 5% milk in TBS-T for 1 h at room temperature. After washing three times, the membrane was covered with Luminata Crescendo (WBLUR0100, Sigma-Aldrich) and the signal was detected with VWR Genoplex. Densitometric analysis was performed with Image J (NIH, US). To test antibodies, the membrane was stripped. After development the membrane was first washed three times for 10 min at RT on the shaker and then incubated for 20 min on the shaker in a solution consisting of Re-Blot Plus Mild (10×) (2502, Merck) diluted with TBS in a ratio of 1:10. The TBS solution was composed of 100 ml 10× TBS and 900 ml distilled water. Before the membrane was incubated with another primary antibody, the membrane was washed twice for 10 min in TBS-T and blocked for 1 h in 5% milk in TBS-T.

#### FACS analysis

For measurement of reactive oxygen species (ROS), we performed FACS analysis. Therefore we seeded PDLF in DMEM high glucose (D5671, Sigma Aldrich), supplemented with 10% FBS (P30-3302, PAN Biotech), 1% AA (A5955, Sigma Aldrich), 1% L-glutamine (G7513, Sigma Aldrich) and 1% ascorbic acid (B6891, Sigma Aldrich). PDLF were stained with chloromethyl-H2DCFDA (C6827, Thermo Fisher) 5.5 h after pressure application. For this purpose, the dye was dissolved in 44.4 µl DMSO (A994.1, Carl Roth) according to the manufacturer’s instructions and 2 µl were pipetted into each well (final dilution 0.1%). After six hours of compressive force treatment, cell culture supernatant was removed and PDLF were solved with 500 μl 0.25% trypsin/EDTA (T4049, Sigma-Aldrich) per well and transferred to FACS tubes. PDLF from three wells were pooled for analysis. Cells were centrifuged at 300 g, 4 °C for 5 min and the supernatant was discarded. After adding 3 ml of PBS, the tubes were centrifuged again with the same settings, the supernatant was discarded and 200 μl of PBS were pipetted onto the cell pellet. Finally, the cell suspension was vortexed and measured with the BD FACSCanto II with a 488 nm solid state 20-mM laser (BD Biosciences; excitation/emission: 492-495/517-527 nm). Gating strategy is presented as Additional file 1: Figure S3.

#### Statistical methods

Symbols represent individual measurements, the horizontal line represents the calculated mean and the vertical line shows the standard error. Statistical evaluation was carried out with GraphPad Prism Version 9.0 (GraphPad software). Normal distribution of the data was first checked using the Shapiro–Wilk test and homogeneity of variance with Levene’s test. Either a Welch-corrected ANOVA with Games-Howell post hoc tests in case of a violation of requirements for parametric testing or an ANOVA with a subsequent Holm-Sidak's post hoc tests was then carried out. Differences were rated as statistically significant at *p* ≤ 0.05.

## Results

### Impact of *Agac* lysate and tensile strain on protein expression of NADPH oxidase-4 (NOX-4) and nitric oxide synthase-2 (NOS-2)

**Fig. 1:**
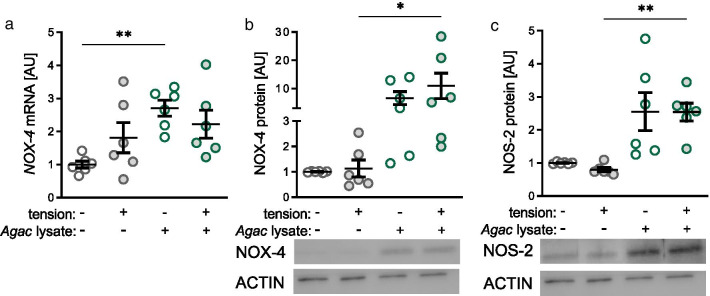
Treatment with *Agac* lysate in combination with tensile strain increased NADPH oxidase 4 (NOX-4) and nitric oxide synthase-2 (NOS-2). Gene (**a**) and protein expression of NOX-4 (**b**) and NOS-2 (**c**) after treatment with *Agac* lysate and tensile strain (cropped blots from Additional file 1: Figure S4); n = 6; *AU* arbitrary units. Statistics: ordinary ANOVA with Holm-Sidak’s post hoc (NOX-4 mRNA and protein) or Welch-corrected ANOVA with Games-Howell post-hoc tests (NOS-2); *p ≤ 0.05; **p ≤ 0.01

After assessment of cell number and lactate dehydrogenase (LDH) release, which revealed a cytotoxic effect of *Agac* lysate and mechanical strain (Additional file 1: Figure S2), we investigated the effects of *Agac* lysate in combination with tensile strain on protein expression of NOX-4 and NOS-2. Tensile strain alone had no effect on *NOX-4* mRNA (*p* = 0.282; Fig. 1a) and protein expression (*p* = 0.964; Fig. 1b). Additional treatment with *Agac* lysate, however, increased NOX-4 expression significantly without tensile strain on mRNA level (*p* = 0.008; Fig. 1a) and with tensile strain on protein level (*p* = 0.05; Fig. 1b). Like NOX-4, NOS-2 protein expression was upregulated significantly with *Agac* lysate in combination with tensile strain in PDLF (*p* = 0.004; Fig. 1c).

### Impact of *Agac* lysate and tensile strain on expression of inflammatory genes

**Fig. 2:**
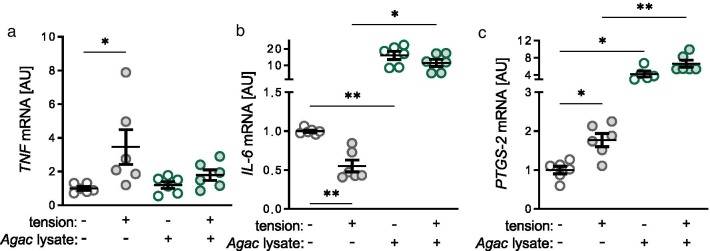
*Agac* lysate increased gene expression of *interleukin-6* (*IL-6*) and *prostaglandin-endoperoxide synthase-2* (*PTGS-2*). Gene expression of *tumor necrose factor* (**a** *TNF*), *IL-6* (**b**) and *PTGS*-*2* (**c**) after treatment with *Agac* lysate and tensile strain; n ≥ 5; *AU* arbitrary units. Statistics: ordinary ANOVA with Holm-Sidak’s post hoc (*TNF*) or Welch-corrected ANOVA with Games-Howell post-hoc tests (*IL-6, PTGS-2*); *p ≤ 0.05; **p ≤ 0.01; ***p ≤ 0.001

Next, we investigated the effect of *Agac* lysate and tensile strain on the gene expression of the inflammatory genes *tumor necrose factor* (*TNF*), *interleukin-6* (*IL-6*) and *prostaglandin-endoperoxide synthase-2* (*PTGS-2*). *TNF* gene expression was upregulated after tensile strain without *Agac* treatment (*p* = 0.020; Fig. 2a). Surprisingly, the effect of tensile strain was inhibited in PDLF treated with *Agac* lysate (*p* = 0.707). *Agac* lysate without tensile strain had no effect on *TNF* gene expression compared to the control group without *Agac* and without tensile strain (*p* = 0.798; Fig. 2a). In contrast to *TNF*, *IL-6* gene expression was reduced with tensile strain in PDLF without *Agac* lysate (*p* = 0.007; Fig. 2b). Treatment with *Agac* lysate increased *IL-6* gene expression without (*p* = 0.006) and with tensile strain (*p* = 0.014) compared to PDLF with no *Agac* treatment (Fig. 2b). Tension elevated *PTGS-2* gene expression (*p* = 0.019) under control conditions without *Agac* (Fig. 2c). With *Agac* lysate *PTGS-2* gene expression was increased without (*p* = 0.026) and with tension (*p* = 0.006) compared to untreated PDLF, while the observed effect of tensile strain was abolished (*p* = 0.171; Fig. 2c).

### Impact of *Agac* lysate and tensile strain on expression of bone remodelling genes

**Fig. 3:**
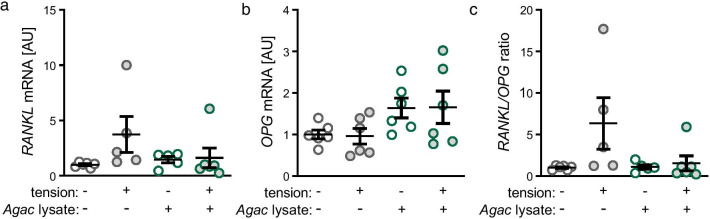
*Agac* lysate in combination with tensile strain did not impact on expression of bone remodelling genes. Gene expression of *receptor of NFk-B ligand* (**a** *RANKL*), *osteoprotegerin* (**b** *OPG*) and *RANKL/OPG* ratio (**c**) after treatment with *Agac* lysate and tensile strain; n = 6; *AU* arbitrary units. Statistics: ordinary ANOVA with Holm-Sidak’s post hoc (*RANKL, OPG*) or Welch-corrected ANOVA with Games-Howell post-hoc tests (*RANKL/OPG* ratio)

Neither treatment with *Agac* lysate (*p* = 0.494) nor tensile strain (*p* = 0.438) impacted on *RANKL* gene expression (Fig. 3a). *OPG* gene expression was also not significantly affected by any tested condition (*p* ≥ 0.234; Fig. 3b). This resulted in an unchanged *RANKL/OPG* ratio after *Agac* lysate treatment without mechanical strain or after tensile strain (*p* ≥ 0.422; Fig. 3c).

### Impact of *Agac* lysate and compression on protein expression of NADPH oxidase-4 (NOX-4) and nitric oxide synthase-2 (NOS-2)

**Fig. 4:**
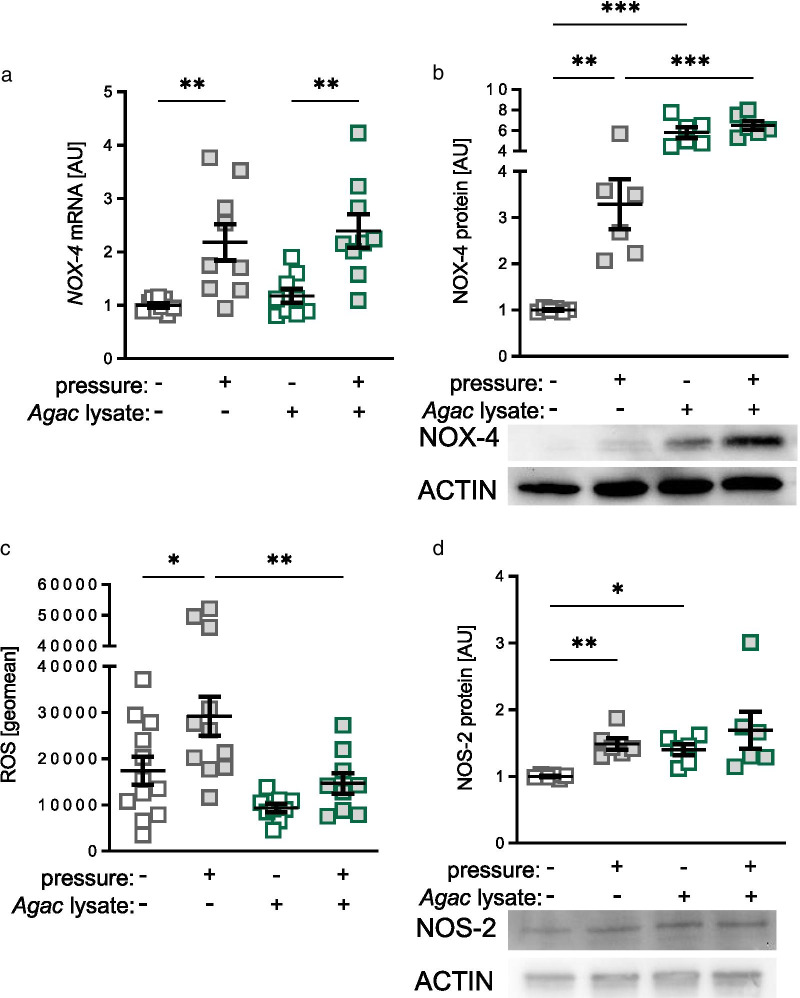
Impact of *Agac* lysate in combination with compressive strain on development of reactive oxygen species (ROS) and expression of nitric oxide synthase-2 (NOS-2). Gene (**a**) and protein expression of NADPH oxidase-4 (**b** NOX-4), development of ROS (**c**) and NOS-2 (**d**) after treatment with *Agac* lysate and compressive strain (cropped blots from Additional file 1: Figure S5); n ≥ 6; *AU* arbitrary units. Statistics: ordinary ANOVA with Holm-Sidak’s post hoc or Welch-corrected ANOVA with Games-Howell post-hoc tests (NOS-2); *p ≤ 0.05; **p ≤ 0.01; ***p ≤ 0.001

Pressure application increased *NOX-4* gene expression without (*p* = 0.004) and with *Agac* lysate (*p* = 0.004; Fig. 4a). *Agac* lysate had no effect on *NOX-4* gene expression under control conditions (*p* = 0.788) and with compressive strain (*p* = 0.788). NOX-4 protein expression was elevated with pressure at control conditions (*p* = 0.003; Fig. 4b). *Agac* lysate increased NOX-4 protein expression without (*p* < 0.001) and with compressive strain (*p* < 0.001) compared to control conditions. The effect of pressure application on NOX-4 protein expression was abolished with *Agac* lysate (*p* = 0.259; Fig. 4b). Under control conditions, pressure application increased ROS development in PDLF (*p* = 0.019; Fig. 4c and Additional file 1: Figure S3). Surprisingly, *Agac* lysate truncated this pressure-induced effect (*p* = 0.008). NOS-2 protein expression was elevated with mechanical strain under control conditions (*p* = 0.008) and after addition of *Agac* lysate (*p* = 0.015; Fig. 4d) without pressure application. With *Agac* lysate, the pressure effect was no longer detectable (*p* = 0.890).

### Impact of *Agac* lysate and compression on expression of inflammatory genes

**Fig. 5:**
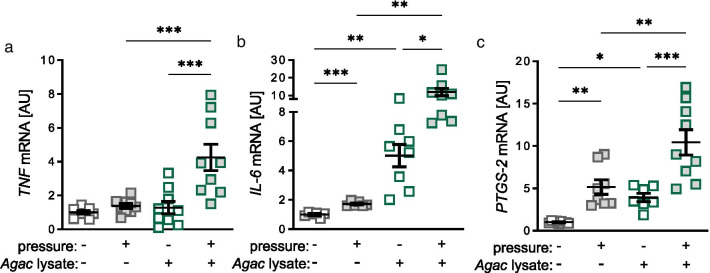
*Agac* lysate in combination with compressive strain increased the expression of inflammatory genes. Gene expression of *tumor necrose factor* (**a** *TNF*), *interleukin-6* (**b** *IL-6*) and *prostaglandin-endoperoxide synthase-2* (**c** *PTGS-2*) after treatment with *Agac* lysate and compressive strain; n ≥ 6; *AU* arbitrary units. Statistics: ordinary ANOVA with Holm-Sidak’s post hoc (*TNF, PTGS-2*) or Welch-corrected ANOVA with Games-Howell post-hoc tests (*IL-6*); *p ≤ 0.05; **p ≤ 0.01; ***p ≤ 0.001

Under control conditions pressure application (*p* = 0.791) or *Agac* lysate treatment (*p* = 0.791) did not significantly impact on *TNF* gene expression (Fig. 5a). However, a combination of mechanical strain with *Agac* lysate elevated *TNF* gene expression significantly compared to compressive force without *Agac* (*p* < 0.001) or *Agac* lysate without pressure application (*p* < 0.001; Fig. 5a). In contrast, *IL-6* gene expression was increased with compression both without (*p* < 0.001) and with *Agac* lysate (*p* = 0.049; Fig. 5b). Treatment with *Agac* lysate elevated *IL-6* gene expression under control conditions (*p* = 0.005) and with pressure application (*p* = 0.007; Fig. 5b). Similarly, we observed upregulated *PTGS-2* gene expression after mechanical strain without (*p* = 0.007) and with *Agac* lysate (*p* < 0.001; Fig. 5c). Addition of *Agac* lysate increased *PTGS-2* gene expression without (*p* = 0.041) and with compressive force treatment (*p* = 0.001) compared to control conditions without *Agac* lysate (Fig. 5c), indicating a boosted inflammatory answer of PDLF to compressive force with *Agac* lysate.

### Impact of *Agac* lysate and compression on expression of bone remodelling genes

**Fig. 6:**
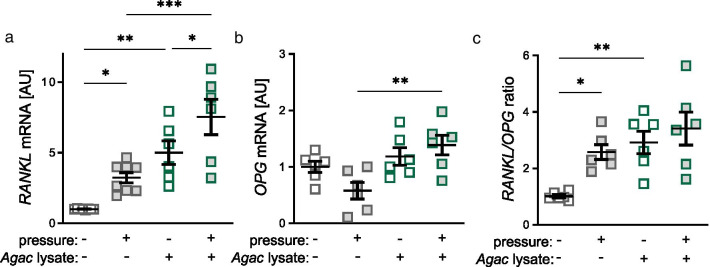
*Agac* lysate in combination with compressive strain increased gene expression of receptor of NFk-B ligand (*RANKL*) and it decoy receptor osteoprotegerin (*OPG*). Gene expression of *RANKL* (**a**), *OPG* (**b**) and *RANKL/OPG* ratio (**c**) after treatment with *Agac* lysate and compressive strain; n ≥ 6; *AU* arbitrary units. Statistics: ordinary ANOVA with Holm-Sidak’s post hoc tests; *p ≤ 0.05; **p ≤ 0.01; ***p ≤ 0.001

For bone remodelling genes, we observed elevated *RANKL* gene expression after compressive force treatment without (*p* = 0.038) and with *Agac* lysate (*p* = 0.038; Fig. 6a). Treatment with *Agac* lysate increased *RANKL* gene expression without pressure (*p* = 0.001) and with compression (*p* < 0.001; Fig. 6a). Surprisingly, *Agac* lysate also increased *OPG* expression with compressive force treatment (*p* = 0.004), while pressure application alone failed to significantly impact on *OPG* gene expression (*p* = 0.158; Fig. 6b). As a result, *RANKL/OPG* ratio increased significantly as expected with pressure application under control conditions (*p* = 0.026; Fig. 6c). Without pressure application, *Agac* lysate upregulated *RANKL/OPG* ratio (*p* = 0.008), while we detected no significant changes with *Agac* lysate combined with mechanical strain (*p* = 0.252) compared to pressure without *Agac* treatment (Fig. 6c). This data indicate, that *Agac* lysate impact on bone-remodelling genes by increasing *RANKL* expression, thereby eventually stimulating bone resorption.

## Discussion

The aim of this work was to elucidate the influence of mechanical compression or isotropic stretching on the expression of inflammatory and bone-remodelling genes in PDLF during mechanical loading, in particular with regard to the development of reactive oxygen species (ROS) and nitric oxide in the presence of lysate of the periodontal pathogen *Aggregatibacter actinomycetemcomitans* (*A. actinomycetemcomitans, Agac*). Our results imply that biomechanical stress is a crucial factor in the regulation of cytokine production and that different inflammatory mediators respond differently to mechanical strain with a potentiating effect by *Agac* lysate.

Various cell populations are contained within the periodontal ligament including fibroblasts, monocytes and macrophages, T cells [27], undifferentiated mesenchymal cells, osteoblasts, osteoclasts and cementoblasts [28], which were subjected to mechanical stress induced by orthodontic treatment. PDLF, which comprise the majority of cells in the periodontal ligament, act as key players during orthodontic tooth movement [9, 10]. At the molecular level, the cells in the periodontal ligament react to mechanical stress with a release of prostaglandins and cytokines, which determine the activity of bone cells [29]. Within the compression areas, the release of inflammatory cytokines such as interleukin-6 (IL-6) and tumour necrosis factor (TNF) [7, 30], the upregulation of prostaglandin-endoperoxide synthase-2 (PTGS-2) as a key enzyme of prostaglandin synthesis [7, 24, 29, 29, 31–33], as well as enhanced nitric oxide generation by NOS-2 [7] by PDLF ultimately trigger the secretion of receptor activator of NF-kB ligand (RANKL) and thus promote osteoclastogenesis [29, 31]. Osteoprotegerin (OPG) acts as an antagonist of RANKL and counteracts osteoclastogenesis [34]. All the described processes apply to a healthy state of the dentition. However, pathological processes in the periodontally challenged dentition create altered conditions that must be taken into account in the context of orthodontic treatment.

The application of orthodontic forces initiates a sterile inflammation in the periodontium [9]. One of the first reactions to mechanical strain is an increased prostaglandin synthesis [31]. PTGS-2 was significantly upregulated by under pressure and stretch conditions. *Agac* lysate provided an additional potentiation of this effect. The production of proinflammatory cytokines by PDLF under mechanical compression is already regulated in the early phase of orthodontically induced tooth movement [24]. In agreement with results of previous in vitro studies, pressure conditions upregulated *PTGS-2* by PDLF with enhanced effects under pre-stimulation with *Agac* lysate [35, 36]. In contrast, an upregulation of gene expression could only be observed for *IL-6* after compression, but not after tensile strain [25]. However, a bacterial stress factor leads to a higher gene expression of *IL-6* for both forms of force application. While the gene expression of *IL-6* and *PTGS-2* in PDLF is dependent on the duration of mechanical compression, a comparable effect seems to occur for *TNF* only in combination with *Agac* lysate and under stretching conditions alone. Although a significant enhancing effect was observed in the stretch control, the bacterial lysate did not appear to potentiate the expression of *TNF* in stretched PDLF. *Agac* even showed tendencies to suppress the gene expression of *TNF* after tensile strain. Although some studies point to an increased synthesis of TNF in orthodontic compression and tensile zones as an initiator of the local inflammatory response in vivo [37–39], our gene expression results showed only an upregulation in response to tensile strain. Stimulation with *Agac* lysate increased gene expression of *TNF* in PDLF after compression. Based on the fact that *TNF* gene expression was significantly upregulated in macrophages after pressure application [40], our results suggest that *TNF* gene expression might be increased at an earlier point in time. Another possible explanation would be that TNF synthesis is up- and down-regulated in a species-specific manner. This hypothesis is supported by different TNF levels measured in gingival sulcus fluid in humans [41] and unchanged mRNA levels in animal experiments [30]. The joint upregulation of *IL-6* and *RANKL* by PDLF under static compression suggests the induction of bone-resorbing processes in the orthodontic pressure zone. During orthodontic tooth movement, increased RANKL and decreased OPG levels have already been detected in adult gingival sulcus fluid and in response to static compression in vitro, but with unchanged protein data [42]. Another in vitro study reported upregulated RANKL levels with pressure conditions, however, as in the present work, without downregulation of OPG, which was attributed to the heterogeneity of the periodontal ligament [31]. Increased catabolic dynamics at orthodontic compression zones were previously corroborated in vivo via RANKL upregulation [38] and are consistent with our results. Contrary to previous results [43], *RANKL* gene expression was not downregulated after tensile strain in this study. In another recent study, the authors observed a trend for increased *RANKL* gene expression which was then associated with a reduced RANKL secretion [44]. Interestingly, the bacterial stimuli showed opposite tendencies on bone metabolism. In an acute inflammatory stage, such as periodontitis or during the early phase of orthodontic tooth movement, RANKL is not synthesised exclusively by osteoblasts but mainly by immune cells and PDLF [9]. The potentiation of the bone-resorbing effects under static compression by stimulus in the form of *Agac* lysate indicate an enhancement of bone resorption. Based on the results regarding the *RANKL/OPG* ratio, it can therefore be assumed that tooth movement in the periodontally damaged dentition, imitated by the presence of *Agac* lysate, can promote bone-degrading mechanisms. Nitric oxide is considered an early modulator of adaptive bone remodelling processes under mechanical strain [45] and plays a mediating role in bone resorption via influencing osteoclast activity [46]. PDLF responded with an increased protein level of NOS-2 under *Agac* stimulus in combination with compression and a potentiating effect of both stimuli in the tensile group. The results suggest that stretched PDLF are stimulated to produce nitric oxide in response to the bacterial stimulus, while static compression emerges even as a stronger physical stressor to induce NOS-2. NOS-2 thus appears to play a role in both compressive and tensile zones of the periodontal ligament during orthodontic tooth movement, as measured by the in vitro model presented here. The role of nitric oxide as an instrument of host response and its effects on bone metabolism have been controversially discussed by Bast et al. [47]. Thus, both stimulatory-destructive and inhibitory-protective associations exist with increased bone loss after experimentally simulated periodontitis induced by infection with periodontal pathogens [48, 49]. Bone metabolism and inflammatory status can also be influenced by ROS [47]. Elevated levels of ROS are involved in the pathogenesis of most inflammatory diseases [50, 51]. Oxidative stress can arise via many different pathways in the human organism and can be attributed to local or systemic factors during orthodontic tooth movement. These include inflamed periodontal condition [52] and aseptic inflammation in the periodontal ligament as a result of mechanical force application [53]. A comparison of these study results with those of the present work leads to the assumption that application of an orthodontic force can be considered the main inducer of oxidative stress in the periodontal ligament during the early phase of orthodontic tooth movement. Contrary to previous assumptions, the quantitative measurement of intracellularly formed ROS by PDLF did not indicate an increased response to the bacterial stimuli. Since high ROS concentrations can lead to apoptosis of gingival fibroblasts [50], it cannot be ruled out that the amount of ROS produced by PDLF independently led to a lower production of reactive oxygen species. Nevertheless, the results of the ROS measurement suggest that significantly higher ROS generation can be initiated by PDLF within compression zones after application of an orthodontic force. In a study by Nguyen et al. (2019), the presence of *P. gingivalis* lysate resulted in significant upregulation of oxidative stress biomarkers and superoxide anions in PDLF [54]. However, no higher ROS levels could be detected towards the lysate [54]. The significantly decreased levels of ROS under bacterial stimulation could be due to antioxidant protective mechanisms of PDLF. Thus, it is possible that a simultaneous endogenous activation of antioxidant enzymes in vitro in the form of superoxide dismutase, upregulation of catalase or increased activity of glutathione peroxidase [53, 55] could have neutralised the resulting reactive oxygen metabolites. This study implies several limitations that should be mentioned. Due to the use of different plates with different surface coatings for performing experiments with compressive or tensile strain, comparisons between these experiments should be done with caution and are not intended. Furthermore, this could also explain different reaction patterns of PDLF on Agac lysate without mechanical strain. Expression profile of PDLF after treatment with Agac lysate and mechanical loading was mostly investigated at the mRNA level, which does not necessarily correspond to the protein level. The main limitation, however, is that the study is an in vitro study, only analysing one cell type of the periodontal ligament and the lysate of only one periodontitis-related bacterium (Additional file 2).

## Conclusions

The impact of *Agac* lysate on inflammatory (*IL6*, *PTGS2*, ROS, NOS2) and bone-remodelling factors (*RANKL*, *OPG*) was investigated in this study. We showed that *Agac* lysate impacts on the expression of genes and proteins involved in inflammation and bone remodelling as well as ROS formation, when PDLF were exposed to mechanical strain, as occurring during orthodontic tooth movement. Our results imply that the presence of *Agac* in periodontal lesions may have a potentiating effect on inflammation and bone resorption, if orthodontic therapy is performed, with progressive attachment and periodontal bone loss to be expected, if performing orthodontic tooth movement in presence of *Agac*. Further insights into how ROS interfere in oral health may contribute to understanding their role in orthodontic tooth movement based on the initiated local aseptic inflammatory response.

## Supplementary Information

**Additional file 1.** Additional file presents (1) fibroblast growing out of periodontal ligament tissue, (2) cell number and lactatedehydrogenase release, (3) gating strategy for ROS FACS and uncropped western blots for figures 1 and 4 (4,5).

**Additional file 2.** Numeric original data set for data presented in this study.

## Data Availability

Datasets are available as additional file 2.
