# Supplementary material for: Effects of mechanical strain on periodontal ligament fibroblasts in presence of Aggregatibacter actinomycetemcomitans lysate
Source: BMC Oral Health. 2021 Aug 18;21:405. doi: 10.1186/s12903-021-01761-3 (PMC8371899; doi:10.1186/s12903-021-01761-3)
Supplement: Supplementary file 1 — Additional file 1. Additional file presents (1) fibroblast growing out of periodontal ligament tissue, (2) cell number and lactatedehydrogenase release, (3) gating strategy for ROS FACS and uncropped western blots for figures 1 and 4 (4,5). [file 12903_2021_1761_MOESM1_ESM.docx]

**Additional file 1 for**

**Effects of mechanical strain on periodontal ligament fibroblasts in presence of *Aggregatibacter actinomycetemcomitans* lysate**

Agnes Schröder^1^, Julia Stumpf^1^, Eva Paddenberg^1^, Patrick Neubert^2^, Valentin Schatz^2^, Josef Köstler^2^, Jonathan Jantsch^2^, James Deschner^3^, Peter Proff^1^, Christian Kirschneck^1^


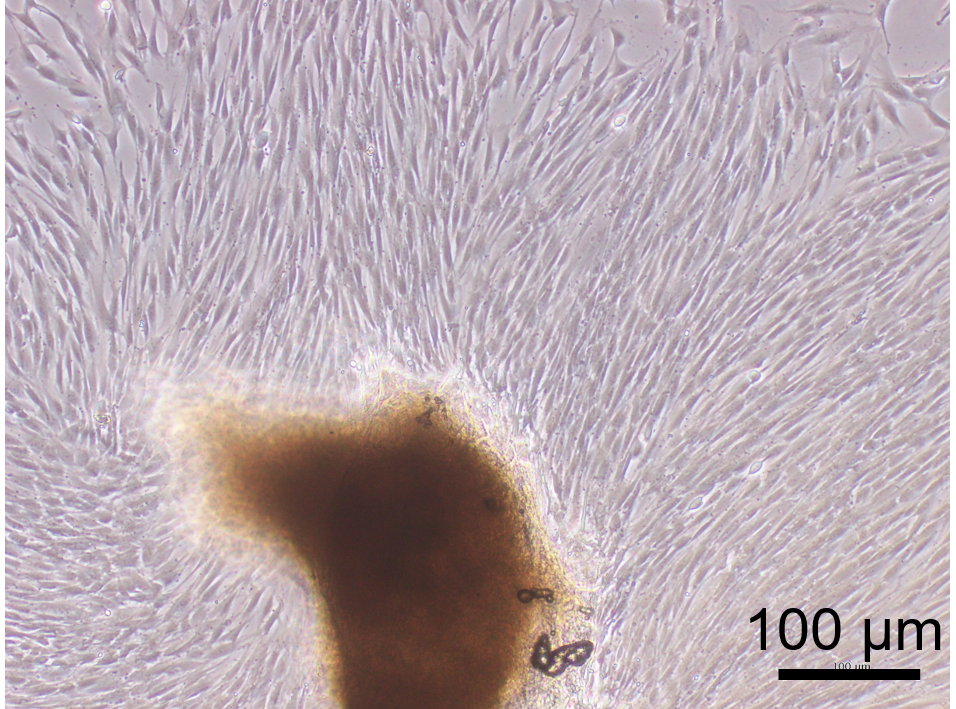


**Additional file 1: Figure S1. Fibroblast growing out of periodontal ligament tissue.** To isolate fibroblasts, periodontal ligament was removed from the middle third of the tooth root under sterile conditions and digested with collagenase type II. Tissue pieces were incubated until fibroblasts grow out.


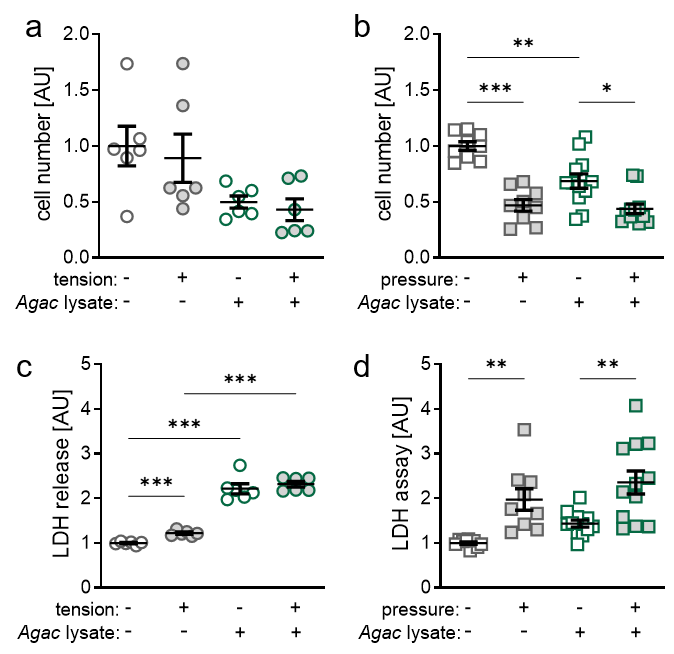


**Additional file 1: Figure S2. *Agac* lysate and mechanical strain reduced number and increased lactate dehydrogenase (LDH) release.** Number of PDLF after tensile strain (a) and compressive strain (b) without or in combination with *Agac* lysate determined with the Z2 coulter particle count and size analyzer (Beckman Coulter) according to manufacturer´s instructions. LDH release after tensile strain (c) and compressive strain (d) without or in combination with *Agac* lysate determined LDH assy according to the manufacturer’s instructions (04744926001, Roche, Penzberg, Germany); n>6; Statistics: (a,d) ordinary ANOVA with Holm-Sidak post hoc or (b,c) Welch-corrected ANOVA with Games-Howell post-hoc tests (NOS-2); *p ≤ 0.05, **p ≤ 0.01; ***p ≤ 0.001.


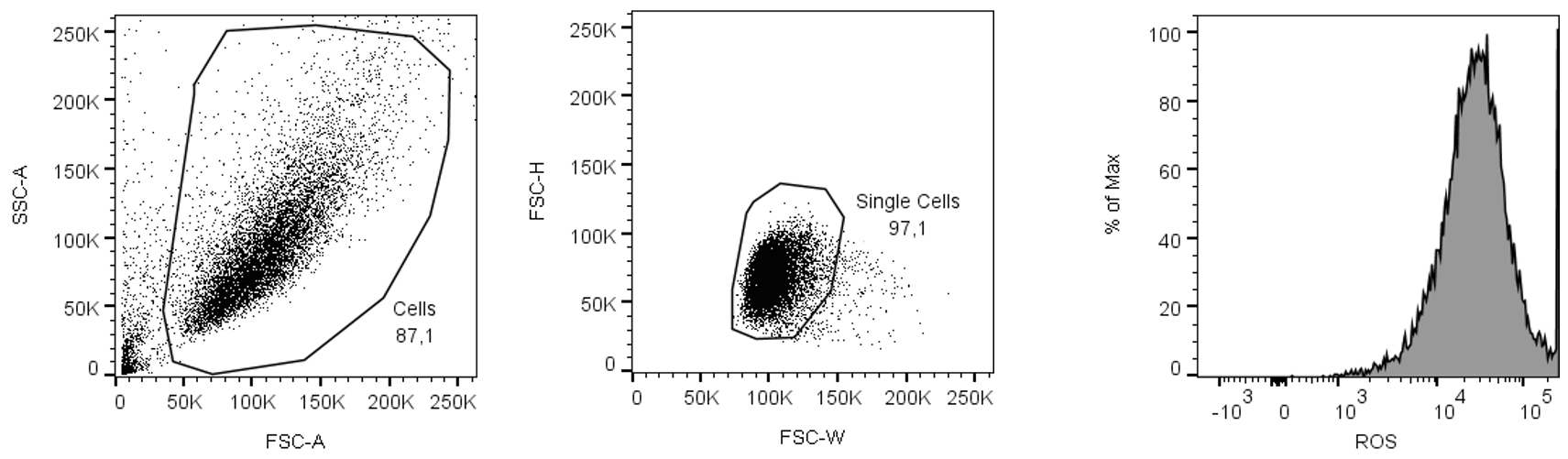


**Additional file 1: Figure S3. Gating strategy for ROS FACS.**


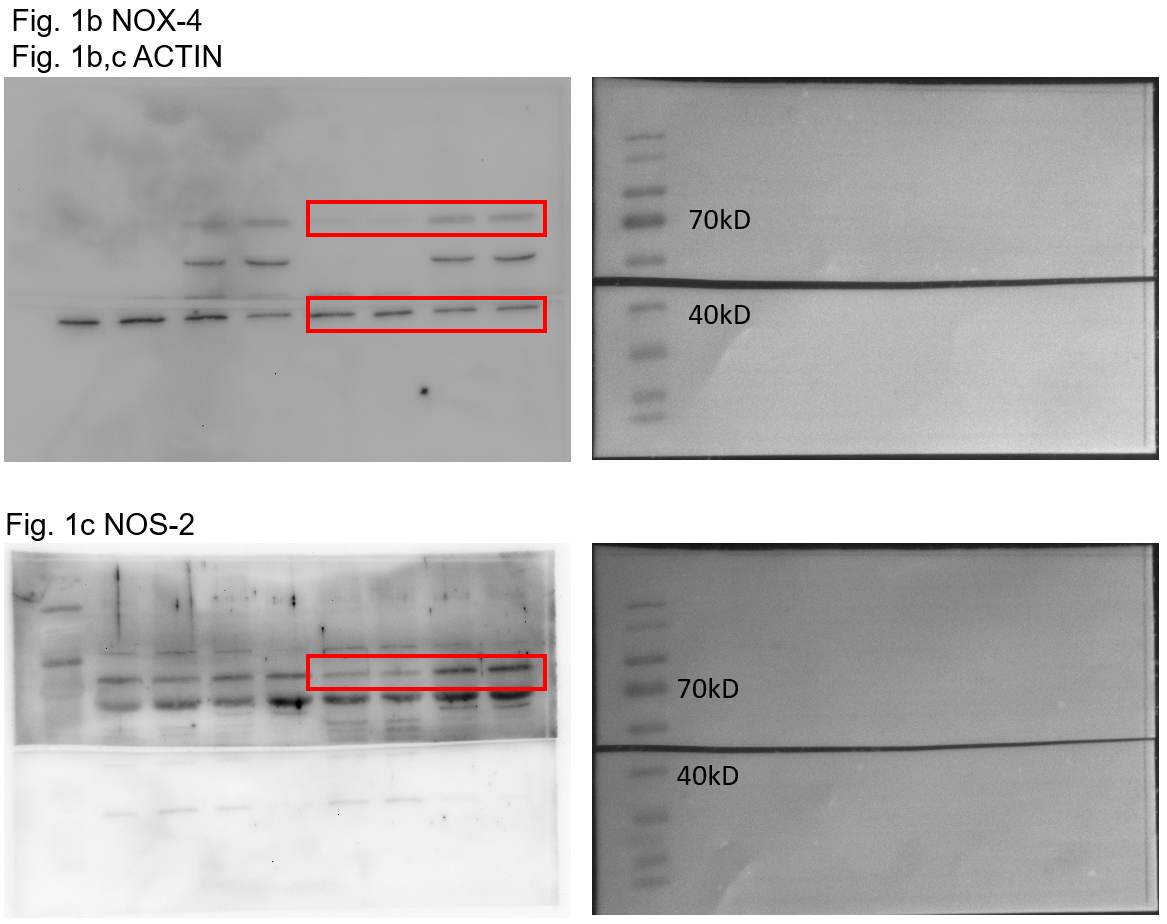


**Additional file 1: Figure S4:** Uncropped blots for Figure 1. The upper part of the blot was stripped and incubated with a second primary antibody.


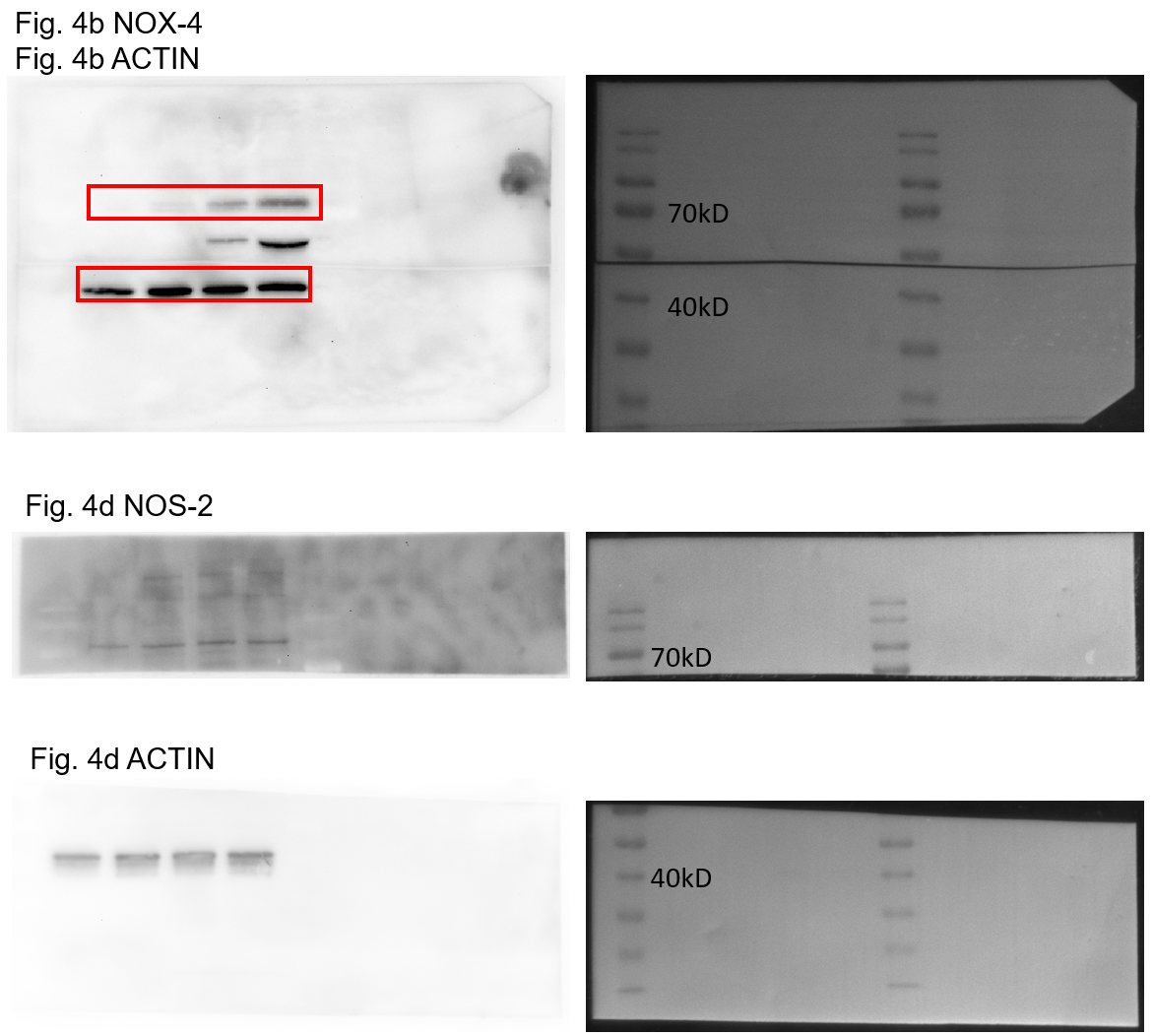


**Additional file 1: Figure S5:** Uncropped blots for Figure 4
