# Supplementary material for: Effects of mechanical strain on periodontal ligament fibroblasts in presence of Aggregatibacter actinomycetemcomitans lysate
Source: BMC Oral Health. 2021 Aug 18;21:405. doi: 10.1186/s12903-021-01761-3 (PMC8371899; doi:10.1186/s12903-021-01761-3)
Supplement: Supplementary file 2 — Additional file 2. Numeric original data set for data presented in this study. [file 12903_2021_1761_MOESM2_ESM.pdf]

Numeric original data set for

Effects of mechanical strain on periodontal ligament fibroblasts in presence of *Aggregatibacter actinomycetemcomitans* lysate

Agnes Schröder[1], Julia Stumpf[1], Eva Paddenberg[1], Patrick Neubert[2], Valentin Schatz[2], Josef Köstler[2], Jonathan Jantsch[2], James Deschner[3], Peter Proff[1], Christian Kirschneck[1]

[1] Department of Orthodontics, University Hospital Regensburg, Germany

[2] Institute of Clinical Microbiology and Hygiene, University Hospital Regensburg, Germany

[3] Department of Periodontology and Operative Medicine, University Medicine Mainz, 55131 Mainz, Germany

Corresponding author: Agnes Schröder

Short title: Effects of *Agac* lysate on PDLF during mechanical strain

Figure 1

Sample	tension	Agac lysate	NOX-4 mRNA	NOX-4 Protein	NOS-2 Protein
1	no	no	1.03	1.02	0.97
2	no	no	1.11	0.96	1.01
3	no	no	0.86	1.03	1.02
4	no	no	0.66	1.02	0.97
5	no	no	1.42	0.98	1.05
6	no	no	0.92	1.00	0.98
7	yes	no	1.66	2.54	0.82
8	yes	no	0.55	0.55	0.73
9	yes	no	1.28	0.45	0.74
10	yes	no	2.80	0.87	0.65
11	yes	no	3.51	1.69	1.10
12	yes	no	1.09	0.69	0.73
13	no	yes	3.35	14.08	1.38
14	no	yes	3.06	6.56	3.57
15	no	yes	1.83	12.93	2.78
16	no	yes	2.66	1.34	1.26
17	no	yes	2.19	3.13	4.76
18	no	yes	3.14	1.63	1.58
19	yes	yes	2.16	28.43	2.37
20	yes	yes	1.23	6.91	3.44
21	yes	yes	1.50	20.86	2.73
22	yes	yes	2.77	2.00	1.43
23	yes	yes	1.66	2.33	2.71
24	yes	yes	4.03	5.15	2.56

Figure 2

Sample	tension	Agac lysate	TNF mRNA	IL-6 mRNA	PTGS-2 mRNA
1	no	no	1.31	0.97	1.02
2	no	no	0.71	0.96	0.87
3	no	no	0.98	1.07	1.10
4	no	no	0.89	0.99	1.14
5	no	no	1.32	1.02	0.60
6	no	no	0.80	1.00	1.27
7	yes	no	4.97	0.47	2.25
8	yes	no	7.88	0.43	2.13
9	yes	no	2.75	0.41	1.88
10	yes	no	1.20	0.73	1.11
11	yes	no	2.09	0.84	1.74
12	yes	no	1.86	0.43	1.52
13	no	yes	1.12	8.48	4.27
14	no	yes	1.51	8.80	3.02
15	no	yes	0.54	18.68	
16	no	yes	1.56	22.36	3.79
17	no	yes	1.77	17.00	3.36
18	no	yes	0.70	20.82	6.77
19	yes	yes	0.86	12.30	9.92
20	yes	yes	2.40	5.32	8.28
21	yes	yes	2.90	5.44	5.39
22	yes	yes	1.95	17.39	5.41
23	yes	yes	1.48	17.14	5.39
24	yes	yes	1.17	11.94	5.48

Figure 3

Sample	tension	Agac lysate	RANKL mRNA	OPG mRNA	RANKL/OPG
1	no	no	1.02	0.94	1.09
2	no	no	1.17	0.91	1.28
3	no	no	0.82	1.15	0.71
4	no	no	1.26	1.39	0.90
5	no	no	0.66	0.63	1.05
6	no	no	1.08	0.98	1.11
7	yes	no	10.00	0.57	17.70
8	yes	no	2.14	0.61	3.48
9	yes	no	3.86	0.48	7.97
10	yes	no		1.15	
11	yes	no	1.43	1.53	0.94
12	yes	no	1.25	1.39	0.90
13	no	yes	0.46	1.33	0.34
14	no	yes	1.95	0.99	1.97
15	no	yes		1.19	
16	no	yes	1.17	1.74	0.67
17	no	yes	1.82	2.02	0.90
18	no	yes	1.88	2.53	0.74
19	yes	yes	6.05	1.03	5.90
20	yes	yes	0.85	0.77	1.11
21	yes	yes	0.93	0.83	1.11
22	yes	yes	0.65	2.58	0.25
23	yes	yes	1.02	3.02	0.34
24	yes	yes	0.25	1.70	0.14

Figure 4

Sample	pressure	Agac lysate	NOX-4 mRNA	NOX-4 Protein	ROS	NOS-2 Protein
1	no	no	0.82	0.96	7877	0.96
2	no	no	1.06	1.00	29450	1.01
3	no	no	1.12	1.04	24029	1.03
4	no	no	1.14	1.05	3503	1.02
5	no	no	0.89	0.96	6506	0.99
6	no	no	0.97	0.99	37159	0.99
7	no	no	0.95		15491	
8	no	no	0.89		12521	
9	no	no	1.16		27839	
10	no	no			13520	
11	no	no			20270	
12	no	no			10773	
13	yes	no	1.70	3.58	49595	1.35
14	yes	no	2.82	2.07	27672	1.30
15	yes	no	3.77	2.23	18118	1.42
16	yes	no	2.54	2.68	11632	1.54
17	yes	no	1.28	3.49	46043	1.87
18	yes	no	1.32	5.68	30843	1.43
19	yes	no	1.75		17703	
20	yes	no	3.52		52078	
21	yes	no	0.94		20212	
22	yes	no			26162	
23	yes	no			21358	
25	no	yes	1.60	6.50	10484	1.12
26	no	yes	1.41	6.31	10916	1.57
27	no	yes	0.89	4.45	9029	1.62
28	no	yes	0.81	7.75	6516	1.42
29	no	yes	1.90	4.99	13832	1.21
30	no	yes	0.90	4.77	4583	1.46
31	no	yes	1.12		8500	
32	no	yes	0.83		9143	
33	no	yes	1.13		11143	
34	yes	yes	4.23	7.52	7909	1.74
35	yes	yes	2.17	6.14	21959	1.29
36	yes	yes	2.83	8.00	7553	3.01
37	yes	yes	1.58	5.30	8826	1.66
38	yes	yes	2.15	6.28	14140	1.30
39	yes	yes	3.23	5.77	12832	1.15
40	yes	yes	0.83		14253	
41	yes	yes	1.12		17384	
42	yes	yes	1.13		27283	

Figure 5

Sample	pressure	Agac lysate	TNF mRNA	IL-6 mRNA	PTGS-2 mRNA
1	no	no	0.96	1.03	0.88
2	no	no	0.92	0.92	0.98
3	no	no	1.12	1.05	1.14
4	no	no	0.92	0.72	0.81
5	no	no	0.62	1.17	1.13
6	no	no	1.46	1.11	1.06
7	no	no	1.24		0.92
8	no	no	0.58		1.21
9	no	no	1.18		0.87
13	yes	no	1.31	1.60	9.01
14	yes	no	1.03	1.66	
15	yes	no	1.11	1.61	5.53
16	yes	no	1.33	1.88	3.63
17	yes	no	1.62	1.59	3.00
18	yes	no	2.15	1.97	3.25
19	yes	no	1.55		4.97
20	yes	no	0.69		8.67
21	yes	no	1.65		3.21
25	no	yes	3.32	5.22	5.29
26	no	yes	1.23	5.65	5.38
27	no	yes	2.48	5.99	4.87
28	no	yes	0.67	2.57	3.17
29	no	yes	0.11	3.57	3.36
30	no	yes	0.25	2.01	1.87
31	no	yes	1.79	8.32	3.48
32	no	yes	1.10	6.79	
33	no	yes	0.56		
34	yes	yes	7.93	10.87	12.50
35	yes	yes	3.91	7.78	5.49
36	yes	yes	3.94	11.72	8.18
37	yes	yes	2.20	10.16	9.00
38	yes	yes	1.51	7.36	14.06
39	yes	yes	2.32	7.24	16.93
40	yes	yes	7.21	24.55	15.68
41	yes	yes	2.94	15.62	7.04
42	yes	yes	6.28		4.93

Figure 6

Sample	pressure	Agac lysate	RANKL mRNA	OPG mRNA	RANKL/OPG
1	no	no	1.00		
2	no	no	1.00		
3	no	no	1.01		
4	no	no	1.00		
5	no	no	1.00		
6	no	no	1.04	0.85	1.23
7	no	no	0.94	1.12	0.84
8	no	no	1.01	1.03	0.98
9	no	no	1.04	1.09	0.95
10	no	no	1.24	1.30	0.95
11	no	no	0.69	0.61	1.13
12	yes	no	4.65		
13	yes	no	3.99		
14	yes	no	3.95		
15	yes	no	2.65		
16	yes	no	3.99		
17	yes	no	2.32	0.93	2.49
18	yes	no	1.95	0.67	2.89
19	yes	no	2.30	1.00	2.30
20	yes	no	1.89	0.52	3.66
21	yes	no	0.44	0.24	1.89
22	yes	no	0.32	0.11	2.97
23	no	yes	5.64		
24	no	yes	7.93		
25	no	yes	6.55		
26	no	yes	3.26	0.81	4.04
27	no	yes	4.03	1.13	3.57
28	no	yes	2.61	1.00	2.61
29	no	yes	2.15	1.48	1.45
30	no	yes	3.21	0.90	3.55
31	no	yes	4.05	1.80	2.26
32	yes	yes	10.93		
33	yes	yes	8.11		
34	yes	yes	9.68		
35	yes	yes	8.91	1.58	5.64
36	yes	yes	4.36	1.05	4.14
37	yes	yes	3.20	1.98	1.62
38	yes	yes	4.78	1.43	3.35
39	yes	yes	2.70	0.76	3.57
40	yes	yes	3.28	1.53	2.14
